# Fine mapping of *yll* prioritizes aberrantly spliced *Glyma.10G090700* (*GmPAO7*) as the strongest candidate in a soybean yellow-leaf lethal mutant

**DOI:** 10.1186/s12870-026-10015-9

**Published:** 2026-09-25

**Authors:** Lei Chen, Jinwei Feng, Jie Ning, Xiaolei Shi, Xintong Wu, Xiangtai Che, Zhuo Li, Zijun Yu, Xin Wang, Qingquan Chen, Haotong Sun, Yanhu Guo, Luxue Liu, Ziqi Ma, Haichao Li, Jing Lin, Jing Zhao

**Affiliations:** 1 School of Life Sciences, Yantai University, Yantai, China; 2 Institute of Cereal and Oil Crops, The Key Laboratory of Crop Genetics and Breeding of Hebei, Hebei Academy of Agricultural and Forestry Sciences, Shijiazhuang, Hebei 050035 China; 3 Institute of Crop Molecular Breeding, Henan Academy of Agricultural Sciences, No.116, Huayuan Road, Zhengzhou, Henan 450002 People’s Republic of China

**Keywords:** Soybean, Yellow-leaf lethal mutant, Candidate gene, Chloroplast, Intron retention, Alternative splicing, Polyamine oxidase, RNA-seq

## Abstract

Chlorophyll-deficient mutants are useful for dissecting chloroplast biology in crops. Here, we characterized a recessive soybean (*Glycine max* (L.) Merr.) yellow-leaf lethal mutant, *yll*, that showed markedly reduced chlorophyll content, impaired photosystem II performance, and seedling lethality. Fine mapping delimited *yll* to a 165-kb interval on chromosome 10. In the Wm82.a6.v1 annotation, this interval contains two genes, of which *Glyma.10G090700* (*GmPAO7*) was prioritized as the strongest candidate on the basis of leaf expression, reduced transcript abundance in *yll*, and a reproducible intron-retention event. Retention of the 110-bp intron between exons 1 and 2 introduced a premature termination codon and was predicted to shorten the annotated 500-aa PAO1-like protein to 32 aa, eliminating the annotated flavin amine oxidase and NAD(P)-binding domains. Direct sequencing of RT-PCR products detected the normally spliced transcript in wild-type plants and the intron-retained sequence in mutant plants, with overlapping splice forms in heterozygous plants; this qualitative assay does not establish splice-isoform proportions. RNA-seq identified broad expression differences between wild-type and mutant sibling seedlings, including enrichment of photosynthesis-, chloroplast-, and redox-associated categories; because *yll* seedlings were severely chlorotic and developmentally compromised, these profiles are interpreted as the transcriptomic state associated with the phenotype and may largely reflect downstream responses. Transmission electron microscopy revealed chloroplast ultrastructural abnormalities associated with *yll*, including poorly developed grana and disorganized thylakoid membranes, but these observations do not functionally validate the candidate gene. Together, the mapping and transcript evidence prioritize *Glyma.10G090700* (*GmPAO7*) as the strongest candidate for *yll*; *Glyma.10G091000* remains in the interval, and functional complementation or an independent allele will be required to establish causality. A PAO–polyamine–ROS connection remains a testable hypothesis because PAO activity, polyamine concentrations, and ROS were not measured.

**Supplementary Information:**

The online version contains supplementary material available at https://doi.org/10.1186/s12870-026-10015-9.

## Background

Soybean (*Glycine max* (L.) Merr.) is a major source of plant protein and oil [1, 2]. Chlorophyll-deficient mutants provide genetic entry points into chloroplast biogenesis and photosynthetic regulation [3–7]. Recent soybean studies illustrate several distinct genetic routes to yellow leaves. The viable yellow-green mutant *jym165* showed reduced pigments, abnormal grana, and altered carbon metabolism but was mapped only to a 3.44-Mb region [8]. The recessive yellow-to-lethal mutant *ytl* was associated with a frameshift in a chloroplast-related PPR candidate within a 418-kb interval [9]. By contrast, *qCC1* is a dominant quantitative locus affecting chlorophyll, photosynthesis, and yield and was associated with an ankyrin-repeat candidate [10]. These studies establish phenotypic and genetic diversity among soybean leaf-color mutants, but lethal chlorosis caused by abnormal processing of a polyamine oxidase transcript has not been described.

Chloroplast development depends on coordinated nuclear and plastid gene expression, protein import, RNA maturation, translation, photosystem assembly, and retrograde signaling [11–16]. Disturbance of these processes can reduce chlorophyll accumulation, damage thylakoid organization, and compromise seedling viability. Chloroplasts are also major sites of reactive oxygen species (ROS) production. Controlled ROS signals participate in acclimation and retrograde communication, whereas excessive ROS can damage membranes and photosynthetic complexes [17–20].

Polyamines, including putrescine, spermidine, and spermine, contribute to development, membrane stability, and stress responses [21–27]. Polyamine oxidases (PAOs) regulate polyamine catabolism or back-conversion and generate H_2_O_2_ as a reaction product [28–35]. A recent genome-wide survey identified 16 soybean PAO-family genes and designated *Glyma.10G090700* as *GmPAO7*; the gene is annotated to encode a 500-aa PAO1-like protein with conserved amine-oxidase-associated features [36]. However, soybean PAO studies have so far been primarily bioinformatic or expression based, and no *GmPAO* has been functionally linked to lethal chlorosis.

Alternative splicing is an important regulatory layer in plants, and intron retention can introduce premature termination codons or promote nonsense-mediated decay [37–39]. Nuclear-encoded factors that control chloroplast RNA splicing are essential for plastid gene expression [40–42], but that process is distinct from spliceosomal processing of a nuclear transcript encoding a plastid-associated or metabolic protein. In legumes, first-intron retention has been reported for a plastid-localized glutamine synthetase transcript in *Medicago truncatula* [43], demonstrating that nuclear transcripts related to plastid metabolism can be alternatively spliced. A direct connection between abnormal splicing of a nuclear *PAO* transcript and chloroplast failure in legumes remains unresolved.

In this study, we characterized the recessive soybean mutant *yll* and integrated fine mapping, physiological measurements, transcript sequencing, and RNA-seq to prioritize *Glyma.10G090700*. We report a stable intron-retention pattern predicted to eliminate the conserved domains of the encoded PAO1-like protein. Because functional validation and direct measurements of PAO activity, polyamines, and ROS are not yet available, we interpret the PAO-redox-chloroplast connection as a testable hypothesis rather than a demonstrated mechanism.

## Methods

### Plant materials

The yellow-leaf lethal mutant *yll* was first observed among progeny of a cross between the soybean cultivars Zheng59 and Luo8C01. Yellow seedlings were visible from emergence and usually wilted and died approximately 10 days after the V3 vegetative stage (three nodes on the main stem bearing fully developed leaves, counting the unifoliolate node). Because homozygous *yll* plants were lethal, the allele was maintained by harvesting phenotypically green plants from segregating heterozygous rows. The phenotype was reproduced under field conditions in Zhengzhou (Henan), Shijiazhuang (Hebei), and Yantai (Shandong), China.

After ten generations of single-seed descent without backcrossing, wild-type and yellow mutant seedlings derived from the same heterozygous progenitor were used as contrasting sibling groups; they are referred to here as WT-sib and *yll*-sib, respectively. WT and *yll* sibling plants were identified from three independent segregating families, designated 24–586, 25–407, and KH467-2. Within each family, WT and *yll* plants were derived from the selfed progeny of the same heterozygous parental plant. These sibling groups were used for physiological assays and RNA-seq. For inheritance analysis, a phenotypically green heterozygous *yll* carrier was crossed with Zao5241 and Jidou17 to generate the ZY and DY populations, respectively. F_1_ plants were selfed separately to produce F_2_ families. The DY population was used for linkage mapping: 22 yellow F_2_ plants were used for the initial genome-wide screen, 537 DYF_2_ plants were used for preliminary localization, and 1,280 progeny plants from selected heterozygous recombinants were used for fine mapping. The *yll* genotypes of recombinant plants were determined by progeny testing in the subsequent selfed generation. For each recombinant-derived line, 40 seeds were sown, and the resulting progeny were scored for segregation of the yellow-leaf lethal phenotype. All materials were grown at the Yantai University experimental field under standard management, with a row spacing of 50 cm and an interplant spacing of 10 cm.

### Phenotyping and genotyping

Leaf phenotypes were photographed at the VC stage, operationally defined here by fully expanded unifoliolate leaves. Total chlorophyll content was quantified in fresh leaves of WT-sib and *yll*-sib plants using 80% (v/v) acetone. For each sample, 0.10 g of fresh leaf tissue, excluding the major veins, was cut into small pieces and immersed in 10 mL of 80% (v/v) acetone in a sealed 15-mL centrifuge tube. Samples were extracted at 4 °C for 24 h in complete darkness, with occasional gentle mixing, until the leaf tissue was completely bleached. The extracts were centrifuged at 5,000 × g for 5 min at 4 °C, and the absorbance of the supernatant was measured at 663 and 646 nm against an 80% acetone blank using a UV-1900 UV–visible spectrophotometer (AOE Instruments, Shanghai, China) equipped with a 1-cm-path-length cuvette. Total chlorophyll concentration (Ctotal, μg mL − 1) was calculated as Ctotal = 7.18A663 + 17.32A646. Total chlorophyll content was expressed as mg g − 1 fresh weight using the equation Ctotal × V × D/(1000 × W), where V is the extraction volume (mL), D is the dilution factor, and W is the fresh weight of the sample (g). For each of the three segregating families (24–586, 25–407, and KH467-2), three independently grown and assayed plants per genotype were used (*n* = 3 plants per genotype per family). The individual plant was the experimental unit for chlorophyll content. Each plant was extracted independently, the three technical absorbance measurements were averaged to obtain one value per plant, and WT-sib versus *yll*-sib was analyzed separately within each family. Thus, nine plants per genotype were measured in total, but they were not pooled into a single *n* = 9 genotype comparison. Chlorophyll fluorescence was measured with an IMAGING-PAM system (Walz, Germany). The reported parameters were Fm', Fo', Y(II), and Fv/Fm. Before measuring the maximum quantum yield of photosystem II (Fv/Fm) using the IMAGING-PAM system (Walz, Germany), leaves were dark-adapted for 30 min. Fresh leaves were collected from WT and *yll* sibling plants derived from three independent segregating families (24–586, 25–407, and KH467-2). For fluorescence parameters, each segregating family was treated as the experimental unit and an independent biological replicate (*n* = 3 families per genotype).

Successful ZY and DY crosses were confirmed using segregating parental markers and progeny-row phenotypes. The *yll* genotype of phenotypically green recombinant plants was inferred by selfing and scoring their progeny for segregation. SSR and InDel genotypes were scored independently of phenotype.

### Fine mapping and candidate-gene analysis of *yll*

For the preliminary linkage screening, 258 SSR and InDel markers distributed across the 20 soybean chromosomes were tested for polymorphism between the DY parents, and informative markers were genotyped in 22 recessive yellow DYF_2_ plants. Markers enriched for the mutant-parent allele were retested in the full mapping population. New regional markers were developed from the Williams 82 reference genome assembly Wm82.a6.v1. Primers were designed using Primer Premier 5. Each marker was first validated on both parents and F_1_ DNA, then on a small segregating panel before population-scale genotyping. Primer sequences are provided in Table S1.

Preliminary linkage analysis used 537 DYF_2_ individuals and placed *yll* between S163 and S752. For fine mapping, heterozygous recombinant F_2_ plants were selfed and 1,280 progeny plants were screened with additional markers. The *yll* genotype of each informative recombinant was assigned by progeny testing in the next selfed generation, delimiting the interval between S560 and S564. Genes in the final interval were retrieved from Wm82.a2.v1 and Wm82.a6.v1. Candidate prioritization considered current annotation support, public tissue expression, WT-sib versus *yll*-sib RNA-seq expression, amplified genomic/cDNA sequence, and transcript structure.

### RNA extraction and transcript analysis

Total RNA was extracted from VC-stage leaves with the RNAprep Pure Plant Kit (Tiangen Biotech, Beijing, China). First-strand cDNA was synthesized using the PrimeScript RT Reagent Kit (TaKaRa, Dalian, China) after DNase treatment with RNase-free DNase I. *Glyma.10G090700* and *Glyma.10G091000* transcripts were amplified with gene-specific primers. Amplicons were resolved by electrophoresis, purified, and subjected to bidirectional Sanger sequencing. Chromatograms were inspected and cDNA sequences were aligned with the relevant genomic annotation to identify splice junctions. Direct chromatogram peak patterns were treated as qualitative evidence and were not used to estimate isoform abundance. Primers are listed in Table S1.

### RNA-seq analysis

VC-stage leaves from WT-sib and *yll*-sib seedlings were collected in three independent biological replicates per genotype. Each biological replicate comprised three individual plants, with one leaf collected per plant; total RNA was extracted separately from each leaf and pooled in equal amounts to generate the biological replicate. RNA integrity was assessed using an Agilent 2100 Bioanalyzer (Agilent Technologies, Santa Clara, CA, USA), and the RIN values ranged from 7.8 to 9.2. RNA-seq libraries were sequenced on an Illumina NovaSeq 6000 platform (Illumina, San Diego, CA, USA) using 150-bp paired-end reads. After adapter trimming and quality filtering, 20.1–25.6 million clean read pairs (5.0–7.5 Gb) were retained per sample, with Q30 values ranging from 94.5% to 96.3%. Of the clean reads, 92.3–95.1% were mapped to the Wm82.a2.v1 reference genome, including 88.7–92.4% uniquely mapped reads. Adapters and low-quality bases were removed using fastp v0.23.4 with the parameters –detect_adapter_for_pe –qualified_quality_phred 20 –unqualified_percent_limit 40 –n_base_limit 5 –length_required 50. Reads were aligned to the soybean Williams 82 reference genome assembly Wm82.a2.v1 using HISAT2 v2.2.1 with the –dta option. The resulting alignment files were converted to BAM format and coordinate-sorted using SAMtools v1.17. Gene-level raw integer counts were generated using featureCounts v2.0.6 with the parameters -p –countReadPairs -B -C -s 0 -t exon -g gene_id. Differential expression was analyzed from raw counts in DESeq2 v1.42.0 in R v4.3.2. FPKM values were used only for descriptive visualization. Genes with |log_2_ fold change|≥ 1 and Benjamini–Hochberg adjusted *P* < 0.05 were considered differentially expressed. PCA was performed on variance-stabilized counts. GO and KEGG enrichment used clusterProfiler v4.10.1 in R v4.3.2. GO annotations corresponding to the soybean Williams 82 reference genome assembly Wm82.a2.v1 were obtained from SoyBase, and KEGG pathway annotations for *Glycine max* (KEGG organism code: gmx) were retrieved from the KEGG database. *P* values were adjusted using the Benjamini–Hochberg procedure, and GO terms or KEGG pathways with an adjusted *P* value < 0.05 were considered significantly enriched. Protein–protein interaction information for DEG-encoded proteins was retrieved from STRING v12.0 using *Glycine max* as the target organism (NCBI taxonomy ID: 3847). Only interactions with a combined confidence score ≥ 0.700 were retained. Proteins that could not be mapped to STRING identifiers were excluded. The resulting PPI network was imported into and visualized using Cytoscape v3.10.2. Accordingly, the statistical n for RNA-seq was three pooled biological replicates per genotype, not nine individual plants.

### Quantitative RT-PCR validation

Ten DEGs were selected for RT-qPCR validation. Primers were designed to span exon-exon junctions where possible and are listed in Table S1. Reactions were performed using a LightCycler 480 Real-Time PCR System (Roche Diagnostics, Mannheim, Germany) with ChamQ Universal SYBR qPCR Master Mix (Vazyme Biotech Co., Ltd., Nanjing, China; Cat. No. Q711). Three independent biological replicates were analyzed for each genotype; each biological replicate consisted of equal amounts of RNA pooled from three leaves collected from three individual plants, and each biological replicate was assayed in three technical replicates. Amplicon specificity was evaluated by melt-curve analysis, which produced a single peak for each primer pair. Amplification efficiencies ranged from 90 to 110% and were calculated as E = (10^(− 1/slope) − 1) × 100%. Relative expression was calculated by the 2^−ΔΔCt^ method only after confirming comparable efficiencies. *GmCYP2* was used as the reference. *GmCYP2* was selected because Jian et al. [44] identified *CYP2* among the most stable reference genes across diverse soybean sample pools and tissues. However, its stability was not formally evaluated between the severely chlorotic *yll*-sib and WT-sib samples in this experiment. The RT-qPCR comparison is therefore used as supportive, direction-of-change evidence, and fold-change magnitudes are interpreted cautiously.

### Statistical analysis

For chlorophyll content, WT-sib and *yll*-sib were compared separately within each segregating family; the independently grown plant was the experimental unit (*n* = 3 plants per genotype per family), and the three technical readings for a plant were averaged before analysis. Because these were family-specific comparisons rather than a pooled genotype analysis, family was not entered as a blocking or random factor; the three families provide independent biological confirmation. For chlorophyll-fluorescence parameters, the segregating family was the experimental unit (*n* = 3 families per genotype). For each two-group physiological comparison, data normality within each group was assessed using the Shapiro–Wilk test, and homogeneity of variance was evaluated using the median-centered Brown–Forsythe test implemented in the car package v3.1–2. Normally distributed data with homogeneous variance were analyzed using an unpaired two-tailed Student’s *t*-test; normally distributed data with unequal variance were analyzed using an unpaired two-tailed Welch’s t-test. Unless otherwise stated, data are presented as the mean ± standard deviation (SD), and *P* < 0.05 was considered statistically significant. Observed segregation ratios were compared with expected Mendelian ratios using Pearson’s chi-square goodness-of-fit test with 1 degree of freedom. Two-sided *P* values, sample sizes, and error-bar definitions are reported with each result. Multiple testing in RNA-seq and enrichment analyses was controlled by the Benjamini–Hochberg procedure.

### Transmission electron microscopy

Fully expanded first trifoliolate leaves were collected from WT-sib and *yll*-sib plants when the first trifoliolate leaf was fully expanded. Three independent plants per genotype were used as biological replicates. Leaf pieces (approximately 1 mm × 1 mm) were immersed in 300 μL of fixative containing 2% paraformaldehyde and 2.5% glutaraldehyde prepared in 0.1 M phosphate buffer (pH 7.2) in 1.5-mL microcentrifuge tubes. Samples were vacuum-infiltrated at 0.1 MPa for 30 min until the tissue pieces sank. Samples were washed three times for 15 min each in 0.1 M phosphate buffer, post-fixed in 1% osmium tetroxide for 2.5 h, and then washed again three times for 15 min each in 0.1 M phosphate buffer. Dehydration was performed sequentially in 30%, 50%, 70%, 80%, and 90% acetone for 15–20 min at each concentration at 4 °C, followed by two changes of 100% acetone for 15–20 min each at room temperature. Samples were infiltrated with acetone:Epon 812 (1:1, v/v) for 3–4 h at room temperature and then with acetone:Epon 812 (1:3, v/v) overnight, followed by pure Epon 812 at 37 °C for at least 5 h. Polymerization was conducted at 37 °C for 12 h and then at 60 °C for 36 h. Ultrathin Sects. (70–90 nm) were prepared using a Leica UC7/FC7 ultramicrotome (Leica Microsystems, Germany), stained with 3% uranyl acetate for 10 min and lead citrate for 2 min, and examined using an HT7800 transmission electron microscope (Hitachi High-Tech, Tokyo, Japan) operated at an accelerating voltage of 80 kV. Representative images were obtained from all three biological replicates.

## Results

### The *yll* mutant exhibits severe chlorophyll deficiency and impaired photosynthetic capacity

**Fig. 1:**
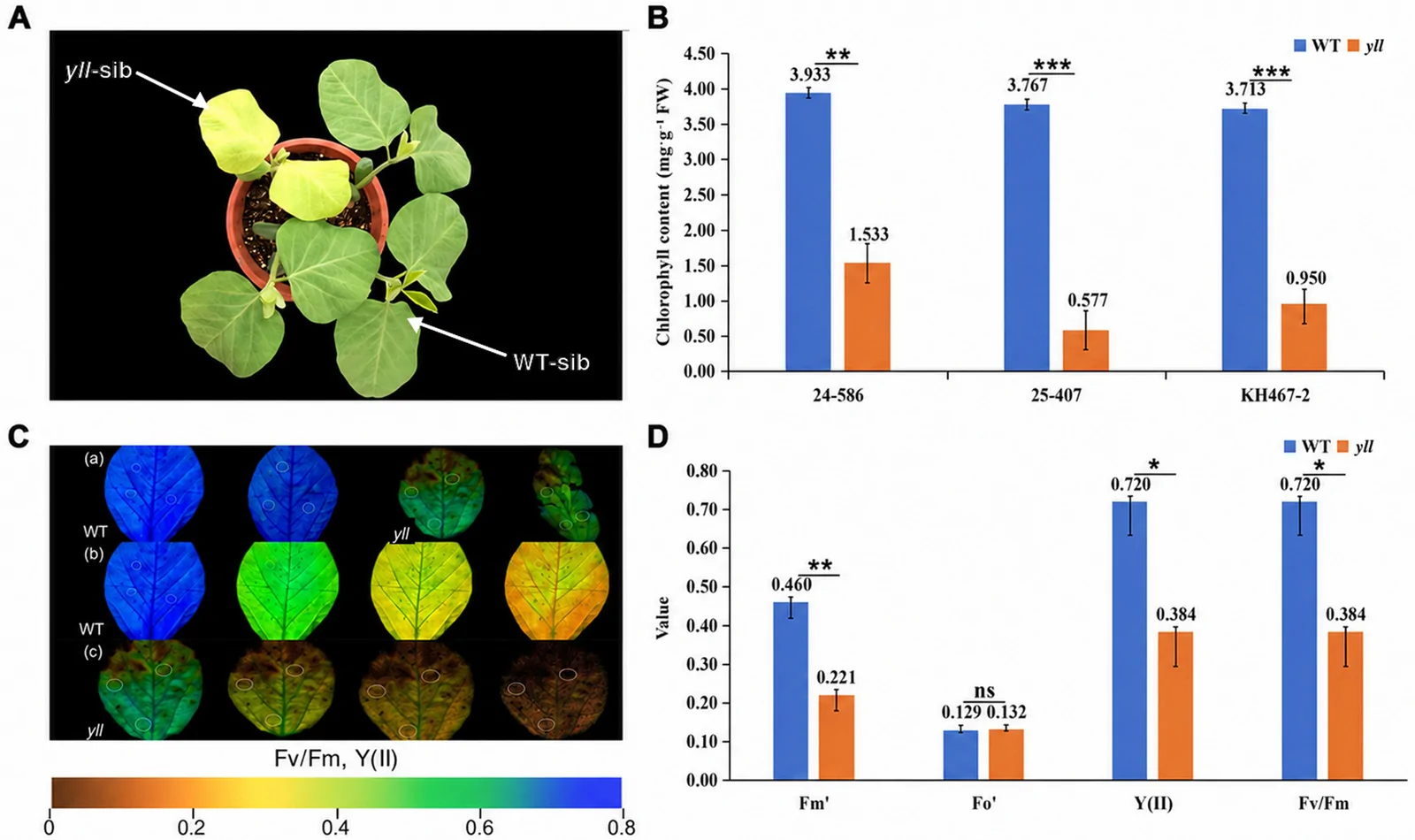
Phenotypic and physiological characterization of *yll*. **A** Representative VC-stage WT-sib and *yll*-sib seedlings photographed under identical conditions. **B** Total chlorophyll content in WT-sib and *yll*-sib plants from three independent segregating families (24–586, 25–407, and KH467-2). Each family was analyzed separately. Within a family, the independently grown plant was the experimental unit (*n* = 3 plants per genotype per family); three technical readings were averaged to obtain one value per plant. Bars show mean ± SE. **C** RGB and chlorophyll-fluorescence images. **D** Fm′, Fo′, Y(II), and Fv/Fm in WT-sib and *yll*-sib leaves. The segregating family was the experimental unit and independent biological replicate (*n* = 3 families per genotype); values are presented as mean ± SE. *P*-value thresholds were calculated using paired two-tailed *t*-tests. *P < 0.05, **P < 0.01, ***P < 0.001; ns, not significant

The *yll* mutant showed severe chlorosis at the VC stage, whereas WT-sib seedlings remained green (Fig. 1A). Mutant plants subsequently wilted and died approximately 10 days after the V3 stage. In the family-specific comparisons shown in Fig. 1B, chlorophyll content in *yll* leaves was approximately 15–40% of the corresponding wild-type value in each of the three segregating sibling families (Fig. 1B).

Chlorophyll-fluorescence images differed visibly between WT-sib and *yll*-sib leaves (Fig. 1C). Fm', Y(II), and Fv/Fm were lower in *yll*, whereas Fo' did not differ significantly (Fig. 1D). These measurements are consistent with impaired photosystem II performance.

### Genetic analysis of the *yll* mutant

A *yll* carrier was crossed with Zao5241 and Jidou17 to generate the ZY and DY populations. All nine F_1_ plants from the ZY cross and all 15 F_₁_ plants from the DY cross exhibited the green phenotype, consistent with the recessive inheritance of the *yll* phenotype.

In the ZYF₂ population, 270 green and 81 yellow plants were observed (*n* = 351), consistent with the expected 3:1 segregation ratio (χ^2^ = 0.6923, df = 1, *P* = 0.4054). Similarly, the DYF₂ population comprised 137 green and 49 yellow plants (*n* = 186), which also conformed to the expected 3:1 ratio (χ^2^ = 0.1792, df = 1, *P* = 0.6721). Thus, the segregation patterns in both populations were consistent with the yellow-leaf phenotype being controlled by a single recessive nuclear gene.

### Fine mapping delimited the *yll* locus to a 165-kb interval on chromosome 10

For preliminary linkage analysis, 258 SSR and InDel markers distributed across all 20 soybean chromosomes were screened using 22 recessive homozygous individuals from the DYF₂ population. Markers showing a high proportion of mutant-parent-like genotypes among these recessive individuals were considered putatively linked to the *yll* locus and were selected for further validation in an enlarged segregating population. Additional polymorphic markers within the linked region were subsequently developed and used for regional mapping. Marker S163 on chromosome 10 showed the strongest linkage signal and was followed by regional marker development.

**Fig. 2:**
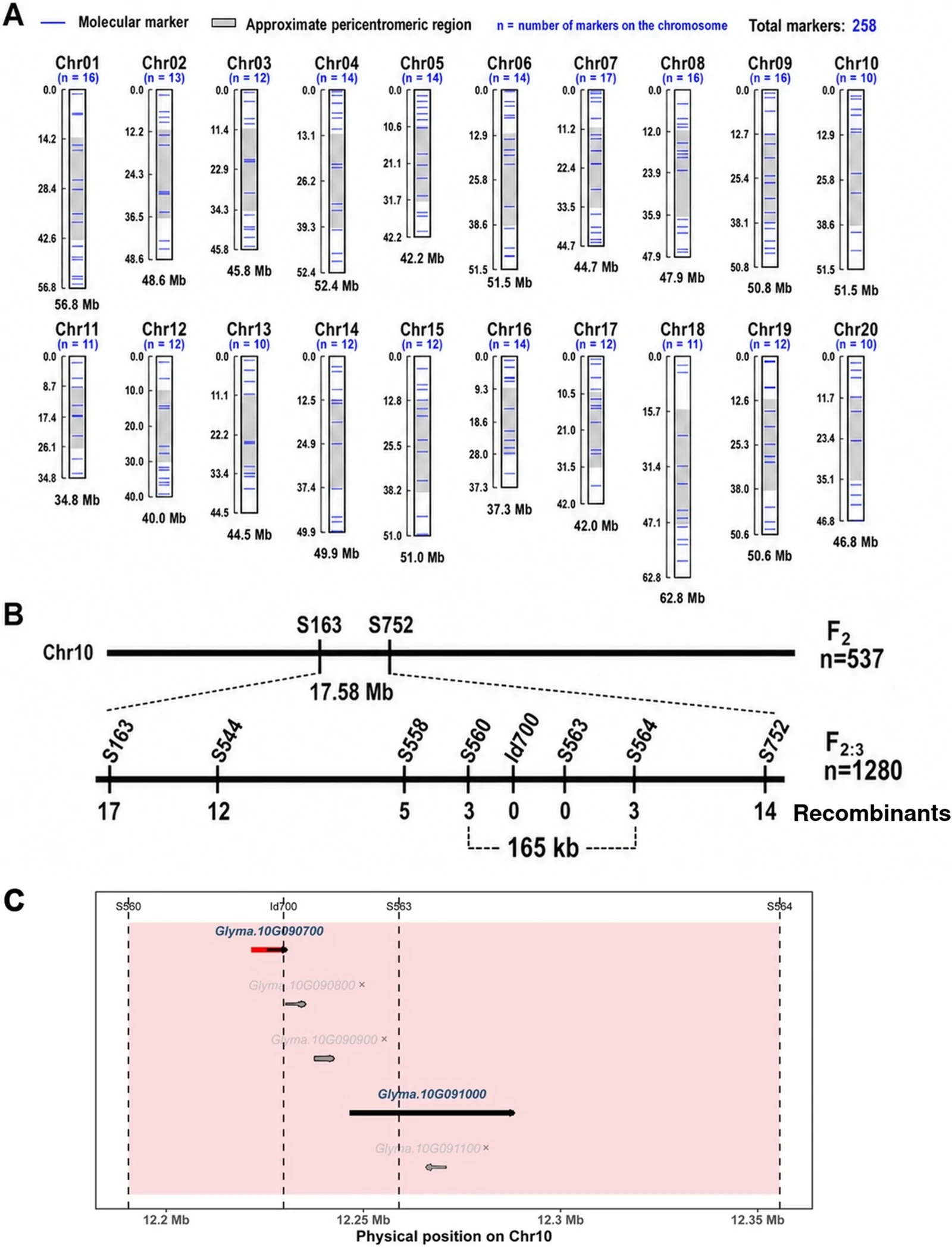
Fine mapping of *yll*. **A** Distribution of 258 markers across the 20 soybean chromosomes. **B** Recombinant mapping of *yll*: preliminary localization between S163 and S752 in 537 DYF_2_ plants, followed by delimitation to the 165-kb S560-S564 interval using 1,280 progeny plants and progeny tests. **C** Gene models in the final interval under Wm82.a2.v1 and Wm82.a6.v1; models not retained in Wm82.a6.v1 are gray. Physical positions are in megabases (Mb)

The *yll* locus was initially mapped between S163 and S752 using 537 F₂ individuals (Fig. 2B). Screening of 1,280 progeny plants from heterozygous recombinants, followed by progeny testing, delimited *yll* to the 165-kb interval between S560 and S564 (Fig. 2B).

Five gene models were present in the Wm82.a2.v1 interval, whereas Wm82.a6.v1 retained only *Glyma.10G090700* and *Glyma.10G091000* (Fig. 2C). The three unsupported older models were not treated as current candidates. *Glyma.10G090700* was prioritized because it showed a reproducible transcript-processing difference between WT-sib and *yll*-sib plants.

### Aberrant splicing of *Glyma.10G090700* (*GmPAO7*) is predicted to severely truncate a PAO1-like protein

**Fig. 3:**
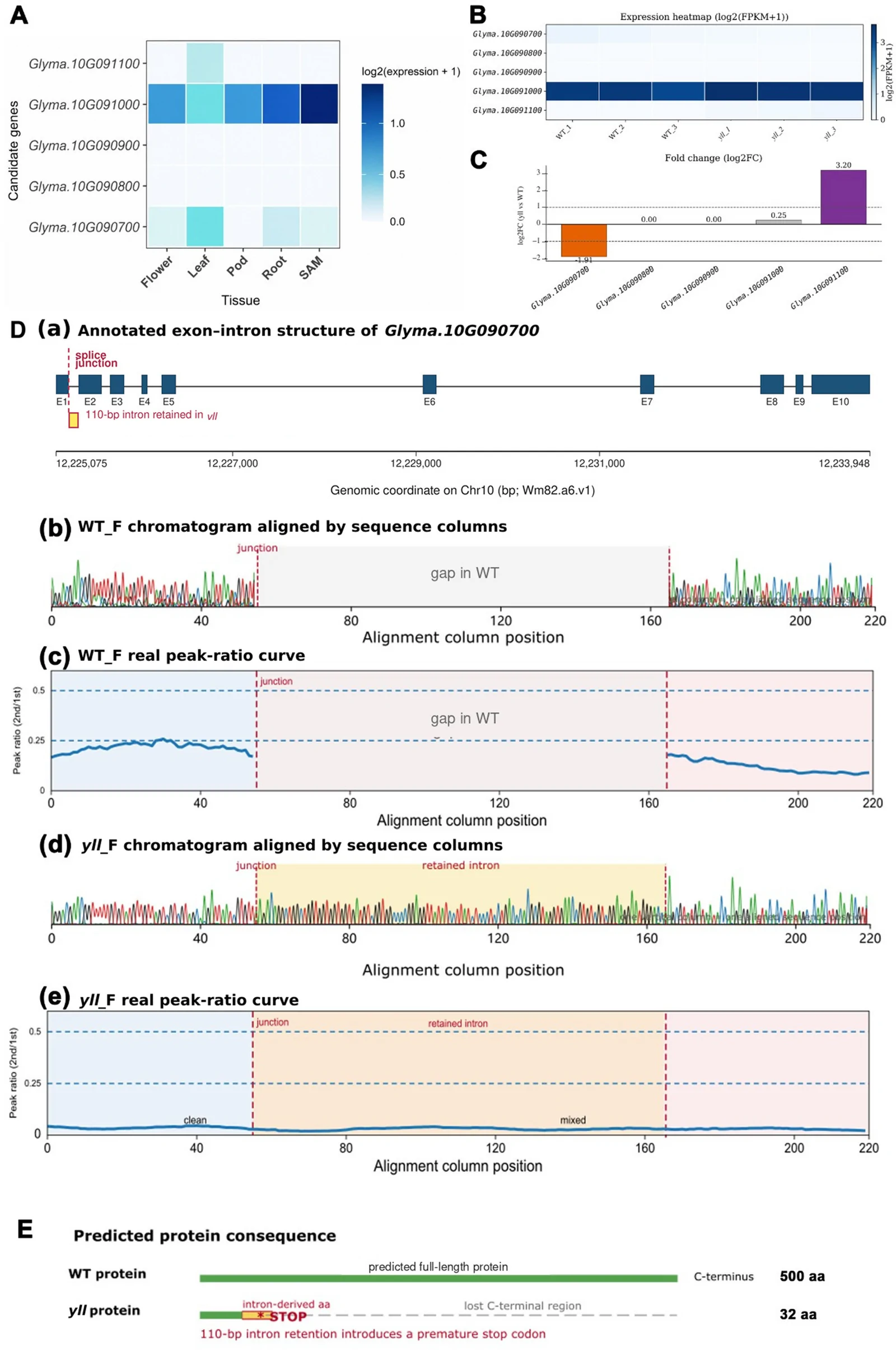
Candidate-gene and transcript evidence in the *yll* interval. **A** Public tissue-expression profiles of the five genes annotated within the *yll* interval in the Wm82.a2.v1 assembly. Public soybean RNA-seq data were retrieved from the SoyBase database and are presented as log_2_(TPM + 1), where TPM denotes transcripts per million. **B** RNA-seq expression in WT-sib and *yll*-sib seedlings. **C** log_2_ fold changes; dashed lines mark |log_2_FC|= 1. **D***Glyma.10G090700* transcript structure and representative splice-junction chromatograms. The 110-bp retained intron between exons 1 and 2 and the premature stop codon are indicated. **E** Predicted full-length (500 aa) and truncated (32 aa) translation products

The Wm82.a6.v1 interval contains *Glyma.10G090700* and *Glyma.10G091000*. Public expression, RNA-seq, amplified sequence, and transcript structure were compared for both genes. Analysis of the public soybean RNA-seq dataset revealed distinct tissue-expression patterns among the five genes annotated within the Wm82.a2.v1 interval. *Glyma.10G091000* was broadly expressed across the examined tissues, whereas *Glyma.10G090700* showed a comparatively leaf-enriched expression pattern (Fig. 3A). In the updated Wm82.a6.v1 assembly, the interval contains only *Glyma.10G090700* and *Glyma.10G091000*. In the present RNA-seq data, *Glyma.10G091000* showed moderate expression in both sibling groups and was not significantly differentially expressed, whereas *Glyma.10G090700* was detectable and lower in *yll*-sib (Fig. 3B, C). No sequence difference or abnormal splice junction was detected in the amplified *Glyma.10G091000* regions. These observations lower the priority of *Glyma.10G091000* but do not constitute formal genetic exclusion.

*Glyma.10G090700* comprises 10 exons and 9 introns and spans an 8,873-bp genomic region at Gm10:12,225,076–12,233,948 on the positive (+) strand of the Wm82.a6.v1 assembly. Predicted translation products of the WT and *yll* alleles comprised a full-length 500-aa protein and a prematurely truncated 32-aa peptide, respectively. No nucleotide difference was detected in the amplified CDS or UTR regions. In *yll*-sib cDNA, the complete 110-bp intron between exons 1 and 2 (Wm82.a6.v1|Gm10:12,225,212–12,225,321 forward) was retained (Fig. 3D). The retained sequence introduced a premature termination codon and was predicted to reduce the translation product from 500 to 32 aa (Fig. 3E). The genomic lesion that causes this splice pattern has not been identified.

Direct Sanger sequencing of RT-PCR products provided qualitative evidence for genotype-associated splice forms. WT homozygotes showed the normally spliced exon 1-exon 2 junction (Fig. S5), whereas *yll* homozygotes reproducibly showed the intron-retained sequence (Fig. S4). Heterozygotes produced overlapping peaks downstream of the splice junction, consistent with co-amplification of normally spliced and retained-intron products (Fig. S3). These chromatograms establish the presence of the genotype-associated splice pattern but do not quantify relative or absolute splice-isoform abundance.

*Glyma.10G090700* was designated *GmPAO7* in a recent soybean PAO-family analysis [36] and is annotated to encode a PAO1-like protein. The annotated 500-aa protein contains flavin amine oxidase and NAD(P)-binding domains, both of which lie downstream of the premature stop codon. Thus, translation of the retained transcript is predicted to produce a 32-aa peptide lacking these annotated domains. Protein accumulation, stability, catalytic activity, and localization were not measured; production of a stable 32-aa peptide and loss of PAO activity therefore remain predictions.

The mapping interval, genotype-associated splice pattern, reduced transcript abundance, and predicted loss of conserved domains together support *Glyma.10G090700* as the strongest candidate for *yll*. Independent functional validation, such as genetic complementation or targeted mutagenesis, will be required in future studies to establish causality.

### RNA-seq reveals broad expression differences between WT and *yll* sibling seedlings

RNA-seq was performed using VC-stage leaves from WT-sib and *yll*-sib plants, with three pooled biological replicates per genotype (*n* = 3); each replicate contained equal amounts of RNA extracted separately from three individual plants. Sample-level relationships were evaluated using PCA and Pearson correlation analysis of DESeq2 variance-stabilized count data. PCA clearly separated the WT-sib and *yll*-sib samples along PC1, which explained 91.78% of the total variance. The WT-sib samples showed greater dispersion along PC2, which accounted for only 6.37% of the variance, while all replicates clustered by genotype, although WT replicates were more dispersed along PC2. Pearson correlation analysis further demonstrated high within-genotype reproducibility: pairwise correlation coefficients were 0.98 among the three *yll*-sib replicates and ranged from 0.95 to 0.96 among the WT-sib replicates. By comparison, correlations between WT-sib and *yll*-sib samples were consistently lower, ranging from 0.92 to 0.93. Hierarchical clustering based on the Pearson correlation matrix independently grouped the three replicates of each genotype into distinct clusters. Although transcriptome-wide correlations were relatively high across all samples, the consistently higher within-genotype correlations and genotype-specific clustering supported the reproducibility of the RNA-seq data. Together, the PCA and correlation results revealed a pronounced transcriptomic distinction between WT-sib and *yll*-sib leaves, consistent with their markedly different physiological and developmental states.

**Fig. 4:**
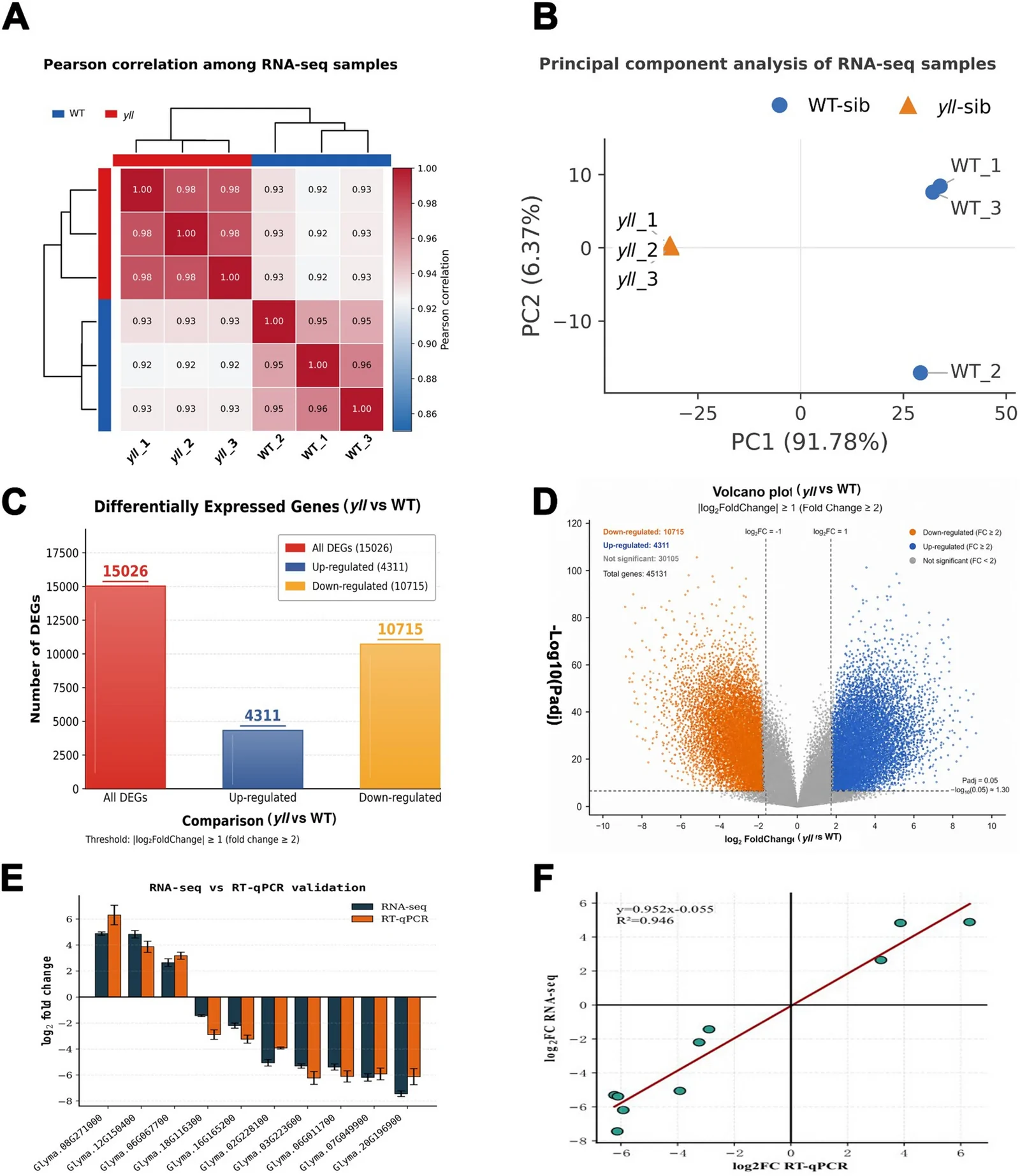
RNA-seq quality assessment and differential expression in WT-sib and *yll*-sib seedlings. **A** Sample-correlation heatmap. **B** PCA of variance-stabilized raw counts from three pooled biological replicates per genotype (*n* = 3); each replicate comprised equal amounts of RNA extracted separately from three individual plants. **C** Numbers of genes with higher or lower abundance in *yll*-sib. **D** Volcano plot; thresholds were |log_2_FC|≥ 1 and adjusted *P* < 0.05. **E** RT-qPCR comparison of ten selected genes. **F** Relationship between RNA-seq log_2_FC and RT-qPCR estimates. Exact n and statistical details are provided in Methods

Using raw-count DESeq2 analysis with |log_2_ fold change|≥ 1 and adjusted *P* < 0.05, 15,026 genes were differentially expressed, including 4,311 with higher and 10,715 with lower abundance in *yll*-sib (Fig. 4). Given the severe chlorosis and developmental arrest, these differences include both potentially proximal responses and extensive secondary consequences.

Under *GmCYP2* normalization, RT-qPCR analysis of ten selected genes showed expression directions broadly consistent with RNA-seq (*R*^*2*^ = 0.946, Fig. 4E, F). Because *GmCYP2* stability was not formally tested between WT-sib and *yll*-sib in this experiment, the RT-qPCR results are treated as supportive evidence for the direction of change rather than as a fully validated quantitative estimate of fold-change magnitude. The selected set included genes annotated for light harvesting, chlorophyll metabolism, starch synthesis, ADP-glucose pyrophosphorylase large subunit, transcription factors, and ADP-glucose pyrophosphorylase subunit (Table S3, Figures S1 and S2).

These results show that severe yellowing in *yll* is associated with widespread transcriptome differences, particularly among photosynthesis-, chloroplast-, metabolism-, and hormone-related genes.

### Functional enrichment highlights chloroplast and metabolic responses in *yll*

**Fig. 5:**
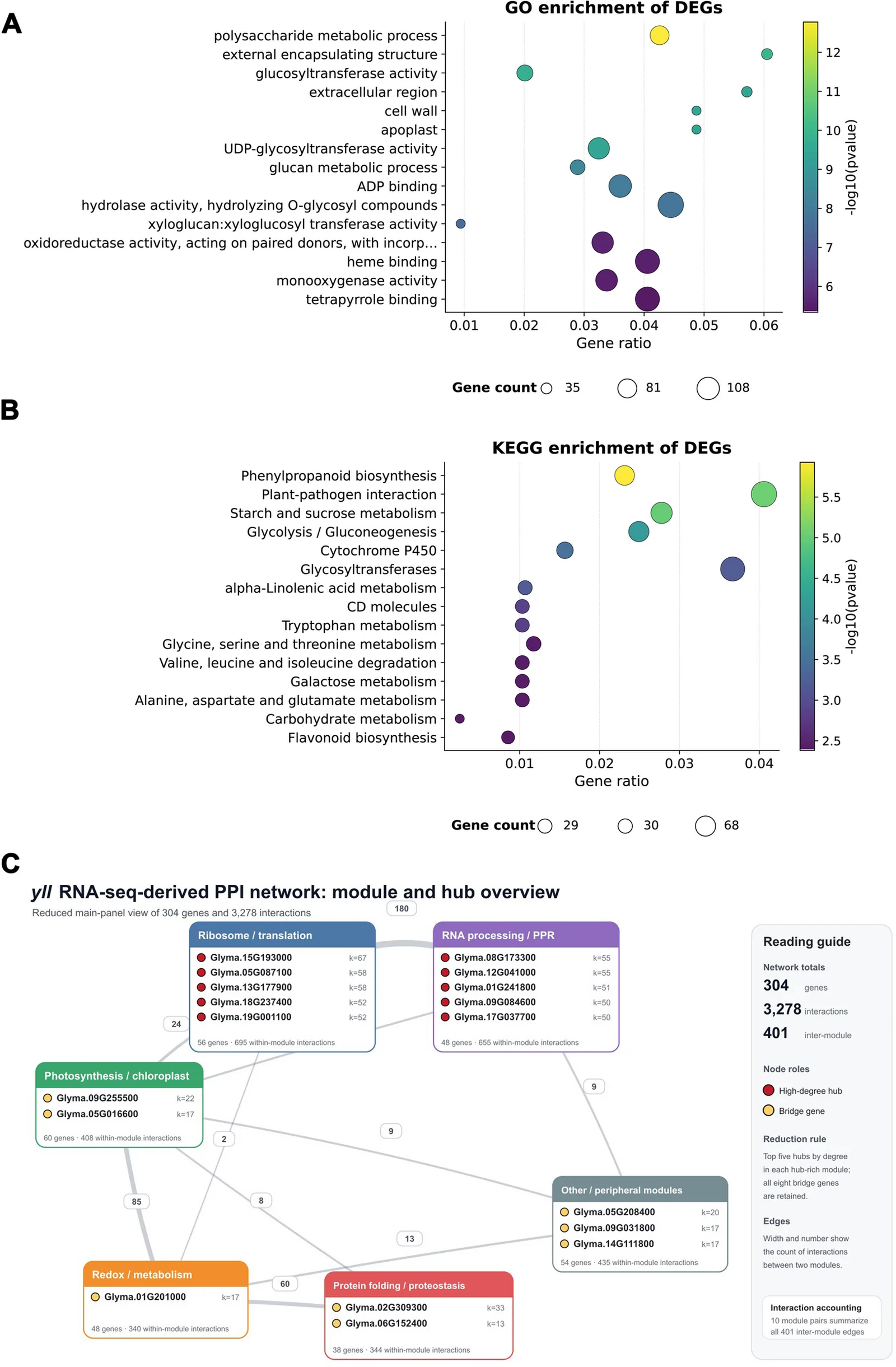
Functional enrichment and interaction-network summaries of genes differentially expressed between WT-sib and *yll*-sib. **A** Gene Ontology (GO) enrichment analysis of DEGs. **B** Kyoto Encyclopedia of Genes and Genomes (KEGG) pathway enrichment analysis of DEGs. In (**A**) and (**B**), dot size represents the number of DEGs assigned to each term or pathway, and dot color represents the − log10(*P* value). **C** Module–hub overview of the reduced PPI network. Boxes represent functional modules. Red and yellow circles indicate high-degree hub genes and bridge genes, respectively. Edge width and adjacent numbers indicate the number of predicted inter-module interactions. The complete network comprised 304 nodes and 3,278 interactions, including 401 inter-module interactions (Figure S6)

GO enrichment of the final raw-count DEG set identified categories related to oxidoreductase activity, tetrapyrrole binding, carbohydrate metabolism, and cell-wall organization (Fig. 5A). These enrichments are consistent with altered photosynthetic and metabolic states.

KEGG analysis identified phenylpropanoid biosynthesis, plant-pathogen interaction, starch and sucrose metabolism, glycolysis/gluconeogenesis, cytochrome P450, and glycosyltransferase pathways (Fig. 5B). These changes are compatible with broad metabolic and stress responses in chlorotic, developmentally compromised seedlings.

A PPI summary grouped selected DEGs into modules related to photosynthesis/chloroplast function, ribosome/translation, RNA processing, redox/metabolism, and proteostasis (Fig. 5C). The network is descriptive and database dependent; it does not demonstrate physical interactions in soybean seedlings or direct regulation by *Glyma.10G090700*.

Overall, the enrichment and network analyses describe the downstream molecular state associated with *yll*. They support chloroplast dysfunction as a major feature of the phenotype but do not establish a direct PAO-polyamine-ROS pathway.

### The *yll* mutant exhibits disrupted chloroplast ultrastructure

**Fig. 6:**
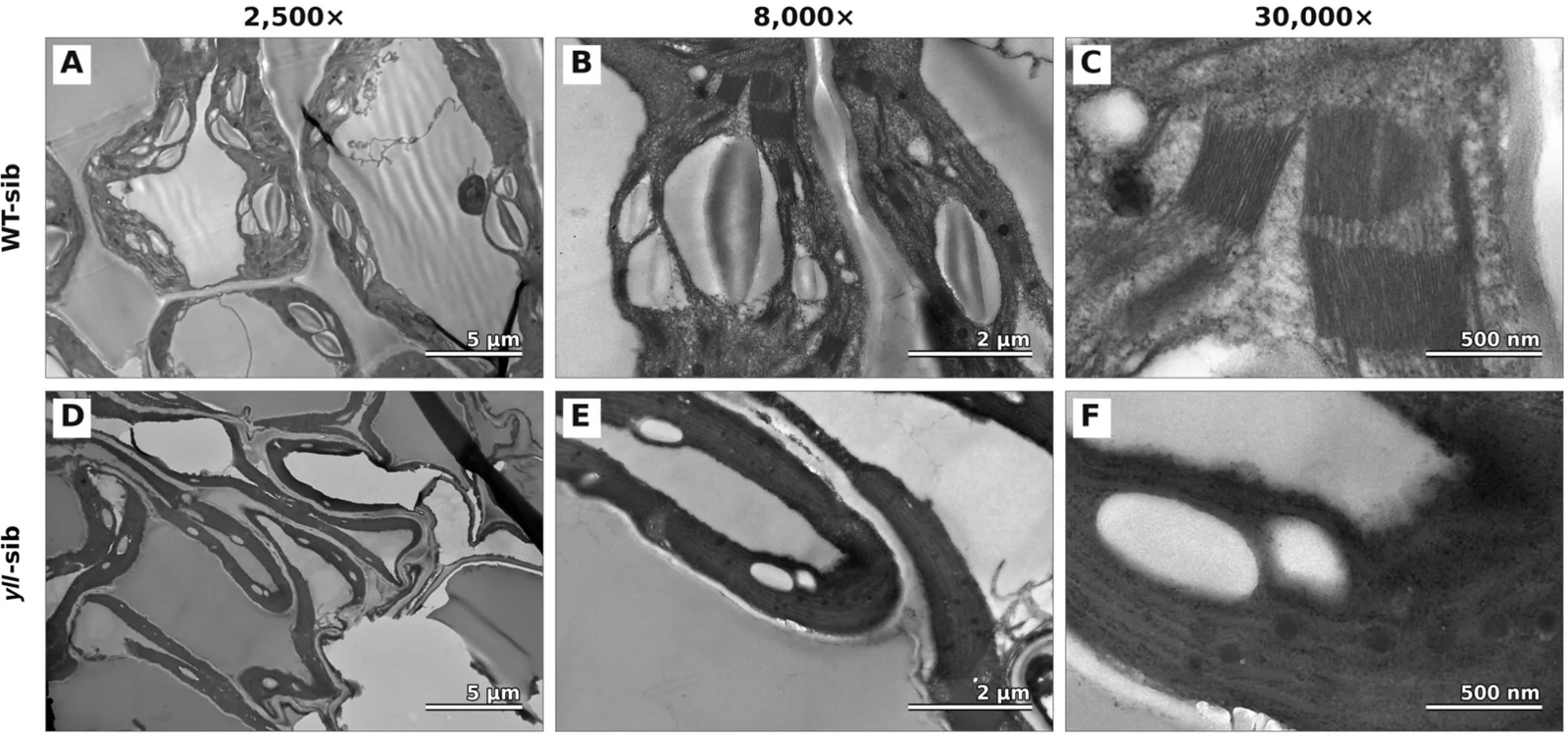
Transmission electron microscopy analysis of chloroplast ultrastructure in WT-sib and *yll*-sib leaves. **A** Low-magnification overview of WT-sib leaf mesophyll cells at 2,500 ×, showing the general cellular organization and chloroplast profiles distributed along the cell periphery. **B** Representative WT-sib chloroplasts at 8,000 ×, showing an intact chloroplast envelope, electron-dense stroma, starch granules, and a well-developed internal membrane system. **C** High-magnification view of a WT-sib chloroplast at 30,000 ×, showing clearly defined, closely appressed grana stacks interconnected by organized stroma thylakoids. **D** Low-magnification overview of *yll-*sib leaf mesophyll cells at 2,500 ×, showing the general organization of mutant mesophyll cells and chloroplast profiles along the cell periphery. **E** Representative *yll*-sib chloroplasts at 8,000 ×, displaying prominent starch granules and a less clearly organized internal membrane system. **F** High-magnification view of a *yll*-sib chloroplast at 30,000 ×, showing poorly defined grana stacks and loosely and irregularly arranged thylakoid membranes. Compared with WT-sib chloroplasts, the representative *yll*-sib chloroplasts exhibited pronounced disorganization of the thylakoid membrane system and impaired grana formation, consistent with the reduced chlorophyll content and photosystem II performance of the mutant. Images were obtained using an HT7800 transmission electron microscope operated at 80 kV. Scale bars = 5 μm in (**A**, **D**), 2 μm in (**B**, **E**), and 500 nm in (**C**, **F**)

To determine whether the yellow-leaf phenotype was associated with alterations in chloroplast ultrastructure, mesophyll cells from WT-sib and *yll*-sib leaves were examined by transmission electron microscopy. At low magnification, WT-sib mesophyll cells displayed generally intact cellular organization, with chloroplast profiles distributed along the cell periphery (Fig. 6A). Chloroplast profiles were also observed in *yll*-sib mesophyll cells, although their morphology and internal organization appeared abnormal (Fig. 6D). At medium magnification, WT-sib chloroplasts exhibited an intact envelope, electron-dense stroma, starch granules, and a well-developed internal membrane system (Fig. 6B). By contrast, the internal membranes of *yll*-sib chloroplasts were less clearly organized, and prominent starch granules occupied a substantial proportion of the chloroplast interior (Fig. 6E). High-magnification observations further revealed clearly defined and closely appressed grana stacks interconnected by organized stroma thylakoids in WT-sib chloroplasts (Fig. 6C). In *yll*-sib chloroplasts, however, grana stacks were poorly defined, and the thylakoid membranes appeared loosely and irregularly arranged (Fig. 6F). These representative ultrastructural observations indicate that the yellow-leaf phenotype of *yll* is associated with pronounced disruption of chloroplast internal membrane organization, consistent with its reduced chlorophyll content and impaired photosystem II performance. These TEM observations characterize abnormalities associated with the *yll* phenotype and do not constitute functional validation of *Glyma.10G090700* (*GmPAO7*).

## Discussion

### Candidate-gene evidence in the context of soybean yellow-leaf genetics

Soybean yellow-leaf phenotypes can arise from defects in chloroplast protein import, photosystem assembly, pigment metabolism, or broader quantitative regulation. Recent examples differ from *yll* in both severity and genetic architecture: *jym165* is viable and mapped to a 3.44-Mb region [8]; *ytl* is seedling lethal and associated with a PPR-gene frameshift [9]; and *qCC1* is a dominant quantitative locus associated with chlorophyll and yield [10]. *yll* is a recessive lethal phenotype mapped to 165 kb. Within the updated annotation, *Glyma.10G090700* is prioritized over *Glyma.10G091000* because it combines leaf expression, reduced mutant transcript abundance, a genotype-associated intron-retention pattern, and a predicted severe truncation. However, prioritization is not proof of causality. *Glyma.10G091000* has not been eliminated genetically, and complementation, knockout, an independent allele, or allelism testing remains necessary.

The study therefore advances *yll* from a phenotype to a tightly mapped locus with a mechanistically plausible transcript defect, while retaining a clear distinction between association and functional confirmation.

### PAO-associated redox regulation is a testable hypothesis

*Glyma.10G090700* (*GmPAO7*) belongs to a 16-member soybean PAO family [36]. Plant PAOs can alter polyamine pools and generate H_2_O_2_, linking polyamine catabolism with developmental and stress signaling [28–35, 45–48]. Loss of the predicted catalytic domains could therefore affect polyamine/redox homeostasis. Nevertheless, *yll* polyamine concentrations, PAO activity, H_2_O_2_, superoxide, antioxidant enzymes, lipid peroxidation, and oxidative damage were not measured. The observed chlorosis and redox-related transcript enrichment are compatible with, but do not demonstrate, a PAO-redox mechanism.

A direct test will require biochemical profiling in developmentally matched WT and *yll* tissues, ideally before severe chlorosis creates widespread secondary stress. Rescue by a wild-type *Glyma.10G090700* allele would then be needed to link any biochemical changes to the mapped locus.

### Biological significance and unresolved origin of the intron-retention pattern

Intron retention is common in plants and can generate premature termination codons, unstable transcripts, or truncated translation products [37–39, 49–52]. In *Medicago truncatula*, first-intron retention modulates a nuclear transcript encoding a plastid-localized glutamine synthetase [43], showing that plastid-related genes can undergo regulated alternative splicing in legumes. In *yll*, the retained intron occurs in a nuclear PAO-family transcript rather than a chloroplast-encoded group II intron, so the event should not be conflated with defects in plastid intron-splicing factors [40–42]. The stable genotype association is notable, but the responsible genomic lesion is unknown. Possible explanations include an unsequenced splice donor/acceptor or branch-point change, an intronic enhancer/silencer variant, promoter or distant *cis*-regulatory variation, structural variation, or a trans-acting splicing defect. Complete genomic sequencing of the locus and flanking region, junction-specific RT-qPCR, and long-read transcript sequencing are required to distinguish these possibilities.

### Most transcriptome changes are likely downstream responses

The very large DEG set is expected for seedlings with severe pigment loss, impaired photosystem II performance, developmental delay, and impending lethality. Photosynthesis, carbohydrate metabolism, stress, hormone, translation, and proteostasis pathways can all change secondarily when chloroplast function collapses. Accordingly, the present RNA-seq data define the molecular state associated with *yll* but cannot separate primary consequences of the candidate lesion from secondary responses. Earlier developmental sampling, inducible perturbation, rescue lines, and biochemical measurements will be necessary to infer causal order.

### Evidence, limitations and priorities for functional validation

Several limitations should nevertheless be acknowledged. Although fine mapping and transcript analyses consistently prioritize *Glyma.10G090700* (*GmPAO7*) as the strongest candidate for *yll*, *Glyma.10G091000* remains in the final interval, causality has not been established by genetic complementation or an independent allele, and the genomic lesion responsible for the intron-retention event has not been identified. The predicted protein truncation and a possible PAO-related polyamine/redox mechanism await protein-level, splice-isoform, and biochemical validation. The RNA-seq, enrichment, and PPI analyses are descriptive of the mutant-associated transcriptomic state, while the physiological and TEM data characterize the *yll* phenotype; none of these downstream observations functionally validates the candidate gene. The mapping and reproducible genotype-associated transcript evidence provide the basis for candidate prioritization, and the required validation experiments remain important priorities for future work.

## Conclusions

The *yll* locus was delimited to a 165-kb interval on soybean chromosome 10. The combined mapping, leaf expression, reduced mutant transcript abundance, and qualitative genotype-associated intron-retention evidence prioritize *Glyma.10G090700* (*GmPAO7*) as the strongest candidate; *Glyma.10G091000* remains in the interval, and causality is not established. Retention of the 110-bp intron between exons 1 and 2 introduces a premature termination codon and is predicted, on the basis of transcript sequence and gene annotation, to shorten the annotated PAO1-like product from 500 to 32 aa and remove the annotated flavin amine oxidase and NAD(P)-binding domains. The mutant phenotype comprises severe chlorophyll deficiency, impaired photosystem II performance, seedling lethality, and associated chloroplast ultrastructural abnormalities. These TEM observations characterize the phenotype but do not validate the candidate gene. RNA-seq, GO/KEGG, and PPI analyses describe the transcriptomic state associated with severe chlorosis and developmental compromise and do not establish direct regulation by *Glyma.10G090700* (*GmPAO7*). Genetic complementation or an independent allele is required to establish causality, and direct measurements of PAO activity, polyamines, and ROS are required to test the proposed PAO–polyamine–ROS hypothesis.

## Supplementary Information

Supplementary Material 1: Figure S1. Representative differentially expressed transcription-factor genes. Heatmap showing selected differentially expressed MYB, NAC, WRKY, bHLH, ERF/AP2, and bZIP transcription factor genes in three biological replicates of VC-stage WT-sib (WT1–WT3) and *yll*-sib (*yll1*–*yll3*) seedlings. Colors represent log₂(expression + 1)-transformed expression values, with blue and red indicating relatively lower and higher expression levels, respectively. The statistical n was three pooled biological replicates per genotype (*n* = 3); each replicate comprised equal amounts of RNA extracted separately from three individual plants.

Supplementary Material 2: Figure S2. Expression patterns of representative hormone-related differentially expressed genes. Heatmap showing selected auxin-, gibberellin (GA)-, cytokinin (CK)-, abscisic acid (ABA)-, and ethylene-related genes in three biological replicates of VC-stage WT-sib and *yll*-sib leaves. Genes are grouped according to their associated hormone pathways. Values are shown as log₂-transformed DESeq2-normalized counts [log₂(normalized count + 1)], with warmer and cooler colors indicating higher and lower expression levels, respectively. The statistical n was three pooled biological replicates per genotype (*n* = 3); each replicate comprised equal amounts of RNA extracted separately from three individual plants.

Supplementary Material 3: Figure S3. Representative Sanger sequencing chromatogram of *Glyma.10G090700* cDNA from a heterozygous plant. Direct Sanger sequencing of an RT-PCR product spanning the first splice junction of *Glyma.10G090700*. The 110-bp intron corresponds to Gm10:12,225,212–12,225,321 in the Wm82.a6.v1 reference genome (1-based, inclusive coordinates). Overlapping peaks downstream of the splice junction are consistent with co-amplification of the normally spliced transcript, in which Gm10:12,225,211 is joined directly to Gm10:12,225,322, and the intron-retained transcript.

Supplementary Material 4: Figure S4. Representative Sanger sequencing chromatogram of *Glyma.10G090700* cDNAfrom the *yll* mutant. Direct Sanger chromatogram of an RT-PCR product from a homozygous *yll* plant shows retention of the entire 110-bp intron (Gm10:12,225,212–12,225,321; Wm82.a6.v1, 1-based inclusive coordinates) between exons 1 and 2 of* Glyma.10G090700*.

Supplementary Material 5: Figure S5. Representative Sanger chromatogram of a WT homozygous *Glyma.10G090700* cDNA amplicon. Direct Sanger sequencing of an RT-PCR product from a WT homozygous plant shows the normally spliced exon 1–exon 2 junction, in which Gm10:12,225,211 is joined directly to Gm10:12,225,322, with the intervening 110-bp intron removed.

Supplementary Material 6: Figure S6. Complete STRING-derived protein–protein interaction network of differentially expressed genes between WT-sib and *yll*-sib seedlings. The network was constructed using STRING v12.0 with a minimum combined confidence score of 0.700. Following confidence filtering and extraction of the connected core network, 304 nodes and 3,278 predicted interactions were retained, including 401 inter-module interactions. Node colors indicate the assigned functional modules: photosynthesis/chloroplast (green), ribosome/translation (blue), RNA processing/PPR (purple), redox/metabolism (orange), and protein folding/proteostasis (red); gray nodes represent other or peripheral modules. Red nodes denote high-degree hub genes, whereas yellow nodes denote bridge genes. Gray edges represent predicted within-module interactions, and black edges represent predicted inter-module interactions. Gene labels identify selected hub and bridge genes. The interactions are database-derived predictions and do not demonstrate direct physical interactions in soybean seedlings.

Supplementary Material 7: Table S1. Primers used in this study.

Supplementary Material 8: Table S2. RNA-seq gene expression matrix.

Supplementary Material 9: Table S3. RT-qPCR validation of RNA-seq data.

## Data Availability

Primer sequences used in this study are listed in Table S1. The RNA-seq expression matrix and RT-qPCR validation data are provided in Tables S2 and S3, respectively. The original RNA-seq data generated in this study have been deposited in the Genome Sequence Archive database under project number PRJCA063758, and will be publicly available upon publication. Reviewer access will be provided during peer review if required.
