# Supplementary material for: Fine mapping of yll prioritizes aberrantly spliced Glyma.10G090700 (GmPAO7) as the strongest candidate in a soybean yellow-leaf lethal mutant
Source: BMC Plant Biol. 2026 Sep 25;26:1884. doi: 10.1186/s12870-026-10015-9 (PMC13732982; doi:10.1186/s12870-026-10015-9)

WT-sib vs *yll*-sib DEG-derived core PPI network

Connected k-core network: 304 nodes, 3278 edges; 401 inter-module interactions retained

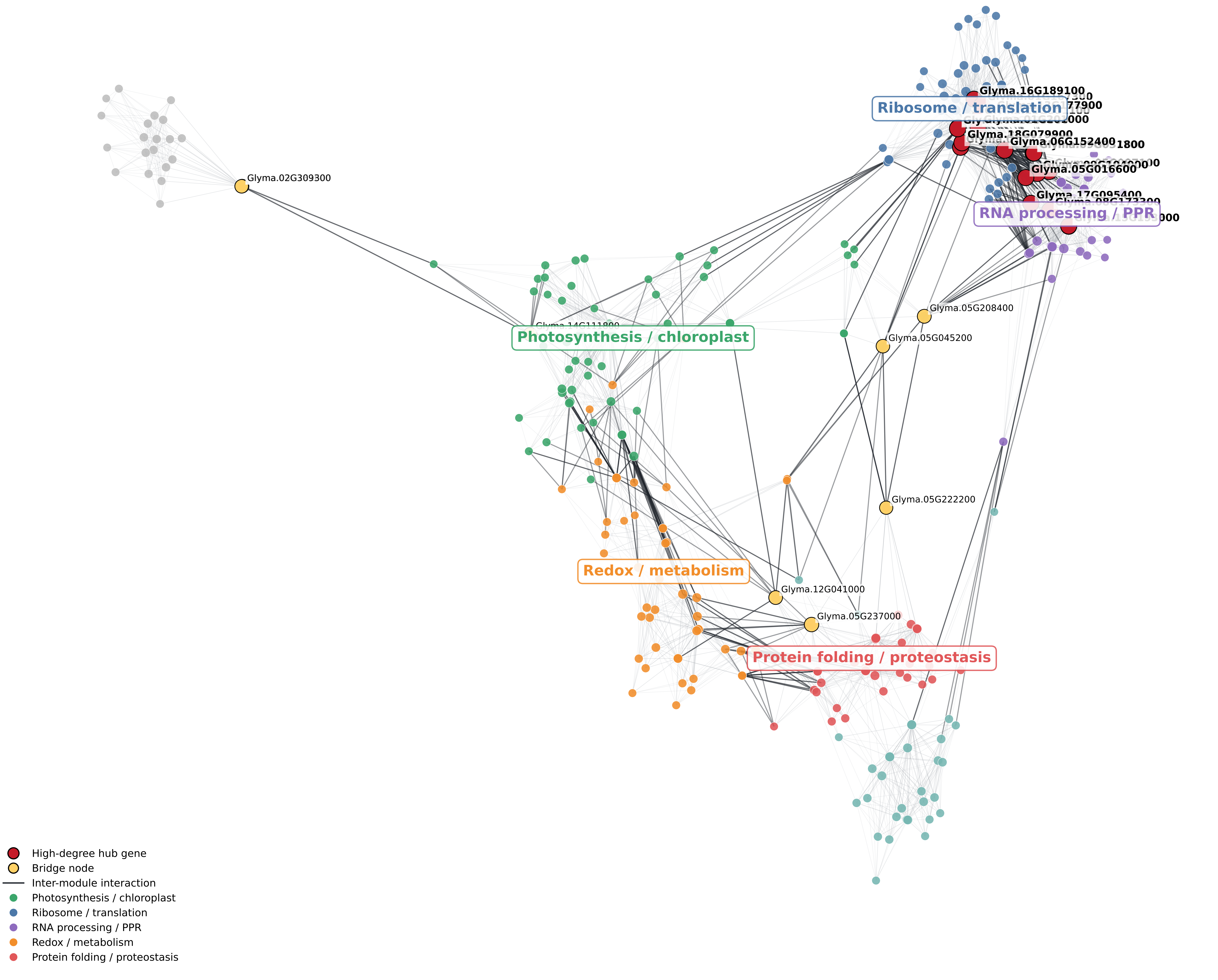

Supplement: Supplementary file 1 [file 12870_2026_10015_MOESM6_ESM.pdf]
